# Gambling among 16-year-olds and associated covariates: A Nordic comparison

**DOI:** 10.1177/1403494820923814

**Published:** 2020-06-10

**Authors:** Jessika Spångberg, Johan Svensson

**Affiliations:** 1 Department of Public Health Science, Stockholm University, Sweden; 2 The Swedish Council for Information on Alcohol and Other Drugs, Stockholm, Sweden

**Keywords:** Adolescent, gambling, problem gambling, ESPAD, cross-country analysis

## Abstract

*Aims*: This study aimed to compare the prevalence in different gambling types as well as problem gambling in the Nordic countries, examining gambling, leisure activities, school truancy, parental relations and consumption of alcohol and other substances as covariates for problem gambling. *Methods*: Cross-country data were provided by the European Survey Project on Alcohol and Other Drugs (ESPAD) 2015. Prevalence of gambling and potential covariates were analysed for Denmark, Finland, Iceland Norway and Sweden (*N*=13,172 respondents aged 16 years), while analyses regarding problem gambling only included countries that participated in the optional questions on gambling problems (Denmark, Finland and Sweden; *N*=8108). We tested variables for problem gambling by bivariate logistic regression and multivariate logistic regression. *Results*: Cross-country differences were found in gambling and problem gambling, as well as differences in covariates for problem gambling. Sweden had the lowest rate of problem gambling. No significant difference was found between Denmark and Finland. Reports of too much gaming, inhalants, slots, betting and online gambling were positively associated with problem gambling, while parental monitoring and parental caring had a negative association. The relevance of the covariates varied across countries. ***Conclusions*: Results indicate that although gambling regulation and its implementation have an important impact on gambling behaviour, we need more research on social, economic and cultural factors and how youth understand and interact with them. Contexts and regulations in other related fields should inform gambling research, policies and interventions.**

## Introduction

Gambling-related harm comprises financial, social and health problems [1,2], co-morbid conditions that have been confirmed through both clinical and epidemiological studies [3–5]. Gambling problems among adolescents are associated with depressive symptoms [6], alcohol and drug problems [7] and delinquent behaviour [8]. Furthermore, early initiation into gambling leads to an increased risk of developing gambling problems later in life [9,10]. Problem gambling is therefore a public-health concern.[1] Nordic countries have to various degrees adopted a public-health approach to problem gambling that is visible in action plans [11,12] and/or by positioning it within gambling regulations or within the agendas of state public-health agencies [13–15].

In the Nordic context, studies examining proportions of problem gambling among adolescents have been based on national prevalence studies using different instruments and conducted in different time periods [16,17], making valid comparisons complicated. Data from the European Survey Project on Alcohol and Other Drugs (ESPAD) 2015 have presented comparisons of the prevalence of gambling among 16-year-olds in Europe, including all the Nordic countries [18,19]. However, significant differences between countries or levels of prevalence of problem gambling have not been presented for 2015, the latest survey. The questions regarding problem gambling have so far been optional in the survey. Therefore, data regarding problem gambling are only available for the Nordic countries that opt to participate: Denmark, Finland and Sweden in 2015 and Denmark and Finland in 2011. In the ESPAD 2011, Denmark had a significantly lower proportion of problem gambling than Finland [20].

This study aimed to compare all the Nordic countries in terms of the prevalence of adolescent participation in different gambling types, as well as the prevalence of problem gambling in Denmark, Finland and Sweden from 2015 ESPAD data.

### Gambling in a Nordic context

The Nordic countries (Sweden, Iceland, Norway, Finland and Denmark) have a common welfare structure including universal health care and education [21]. This is a strong determinant of adolescent health [22]. These countries seem to adhere to a healthier lifestyle, as well as reporting better self-rated health than other European countries [23]. Although the status of the common Nordic welfare regime may have become uncertain due to pressures from globalisation and neoliberalism [21,24], indicators of such welfare structures suggest there are structural commonalities within the Nordic countries.

### Gambling regulation

Regulation in Nordic countries as well as the rest of Europe stems from national prioritisation of needs rather than regulative imperatives from the European Union [25]. Still, the European Commission has launched a non-binding recommendation on consumer protection of online gambling including a series of principles regarding self-restraint measures, problem gambling identification systems and risk information, as well as rules on underage gambling and advertisements [26]. Most gambling regulations stipulate a ban on marketing gambling products towards youth. Still, the implementation varies among countries. A Finnish study as well as a Norwegian study found that younger gamblers (18–24 years) reported stronger impacts on involvement and knowledge from advertisements than older gamblers [5,27].

### License and monopolies

After Denmark dismantled its gambling monopoly in favour of a license-based system in 2012, Sweden launched new regulation of its license-based system in January 2019 [26]. Finnish and Norwegian monopolies of the state gambling operators offer various online games. Online gambling sites require registration and identification of gamblers, whether the online games are offered within a monopoly or a license-based system. In addition, all Nordic countries with regulated online gambling markets have introduced different kinds of player tracking instruments [28]. Further, in Iceland, all gambling is restricted to non-state institutions and charity organisations with a licence [15]. In 2017, the three Finnish gambling monopoly operators merged into a single state-owned monopoly operator, Veikkaus Ltd [5].

### Age limits

Most European countries have adopted an age restriction of ⩾18 years for gambling. In Finland, the age limit for gambling was raised from 15 to 18 years through an amendment to the Lottery Act in 2010. Slot machines were given a transition period with age limits, enforced in 2011 [29]. However, there are differences in policies about and availability of different gambling types. For example, only in Finland are gambling machines accessible in ordinary stores [5], and online gambling for poker and casinos has not been legalised in Iceland [30,31].

### Previous research on adolescent gambling

The male sex, low socio-economic status, depression, poor academic performance, alcohol and substance use and negative parental influences have been identified in longitudinal research as risk factors for problem gambling among youth [6,7]. In examining ESPAD data for Europe, Molinaro found a positive association between youth gambling and areas such as participation in sports, playing video games (‘gaming’), alcohol and substance use and relationships with parents [19]. Reading books for leisure, not easily receiving money from parents and parents’ monitoring of Saturday night activities had a negative relation to gambling, and hence may act as protective factors. These covariates were not tested for problem gambling. Several of the risk factors for problem gambling also appear to be predictors of multiple risk-taking behaviours such as alcohol and drug abuse [32]. We know less about the similarity of these predictors of problem gambling to those in the Nordic countries in particular, since our knowledge is based on national studies that have not examined the same covariates [17].

Earlier studies have called for research focusing on differences and similarities in gambling behaviours among adolescents in the Nordic countries [17]. Exploring potential covariates in Denmark, Finland, Iceland, Norway and Sweden could improve our understanding of factors that are relevant to underage gambling and to problem gambling among adolescents in Nordic countries.

### Aim

This study’s overall aim was to compare the Nordic countries in terms of prevalence of participation in different gambling types as well as problem gambling in Denmark, Finland and Sweden. A further aim was to determine whether gambling types and covariates identified as associated with adolescent gambling in the ESPAD study of 2015 were also associated with problem gambling in Denmark, Finland and Sweden together as well as in each country individually. The covariates reside in the areas of leisure activities, school truancy, relationships with parents, consumption of alcohol and other substances and gambling types.

## Methods

### Data set

Data were provided by the ESPAD, a school-based survey conducted with a large sample of 16-year-old students. The overall aim of the ESPAD is to collect comparable data on substance use among 15- and 16-year-old students in as many European countries as possible. Students’ participation is voluntary and anonymous. Apart from using a common questionnaire on a commonly defined target population and data-collection period, fieldwork practices as well as capture, cleaning, delivery and analyses of the data are standardised. Questions on gambling, both overall and various gambling types, were first included in the ESPAD in 2015. Problem gambling was included as an optional part of the survey in 2011 and 2015. For more information on ESPAD methods, see www.espad.org/.

The analyses on gambling include all five Nordic countries, with 13,172 total respondents (6491 boys and 6682 girls; Table I). For analysis of gambling problems, only countries that took part in the optional questions on problem gambling (Denmark, Finland and Sweden; *N*=8108; 3929 boys, 4179 girls) were included. In the ESPAD, representativeness is shown in national sampling frame and data-collection mode (paper and pen with teachers as survey leaders) that all included countries used. Further, students’ presence rates were high (Denmark 88%, Finland 89%, Iceland 85%, Norway 90% and Sweden 86%). With the exception of Denmark (26%), school participation was high (Finland 85%, Iceland 79%, Norway 53% and Sweden 83%). According to the ESPAD methodology report, there were no indications of bias noted for the net sample, and the Danish team found the collected data representative for Danish students [33].

**Table I.** Leisure activities, school truancy, family environment, substance use, gambling and problem gambling in ESPAD 2015.

		Denmark	Finland	Iceland	Norway	Sweden	Total
Participants (*n* boys, *n* girls)		1654 (788, 866)	3992 (1919, 2072)	2613 (1287, 1326)	2451 (1275, 1176)	2462 (1222, 1240)	13,172 (6491, 6681)
Mean age (years)		15.8	15.8	15.8	15.8	15.7	15.8
Problem gambling	Lie/Bet	8.6 (7.3–10.0)	9.0 (8.1–9.9)			5.4 (4.5–6.3)	7.8 (7.2–8.4)
Leisure activities	Spend too much time gaming	17.2 (15.4–19.0)	16.6 (15.4–17.7)	17.0 (15.5–18.4)	17.5 (16.0–19.0)	23.7 (22.0–25.4)	18.2 (17.6–18.9)
	Active participation in sports	88.5 (87.0–90.0)	95.3 (94.7–96.0)	92.0 (91.0–93.0)	80.2 (78.6–81.8)	88.4 (87.1–89.6)	89.7 (89.2–90.2)
	Read books for enjoyment	22.0 (20.0–24.0)	18.3 (17.1–19.5)	33.1 (31.3–34.9)	20.5 (18.9–22.1)	22.1 (20.5–23.8)	22.8 (22.1–23.5)
School truancy	3 or more days of school missed	25.8 (23.6–27.9)	38.9 (37.3–40.4)	29.5 (27.7–31.2)	21.8 (20.1–23.4)	33.8 (32.0–35.8)	31.3 (30.5–32.1)
Relationship with parents	Can easily obtain money as a gift from parents	21.0 (19.9–22.9)	18.5 (17.3–19.7)	17.4 (15.9–18.8)	12.9 (11.6–14.2)	15.9 (14.4–17.4)	17.0 (16.4–17.7)
	Parental monitoring of Saturday night activities	96.3 (95.4–97.2)	88.0 (87.0–89.0)	92.9 (91.9–93.9)	93.4 (92.4–94.4)	89.6 (88.4–90.8)	91.3 (90.8–91.2)
	Parental emotional support	89.8 (88.4–91.3)	87.9 (86.8–88.9)	86.8 (85.5–88.1)	85.5 (84.1–86.9)	87.2 (85.9–88.5)	88.0 (87.8–88.8)
Substance use	Alcohol use in last month	73.5 (71.4–75.6)	32.1 (30.6–33.5)	9.2 (8.1–10.4)	23.7 (22.1–25.4)	25.7 (24.0–27.4)	29.9 (29.1–30.7)
	Binge drinking in last month	31.9 (29.6–34.1)	12.9 (11.8–13.9)	2.8 (2.2–3.5)	8.6 (7.5–9.7)	9.2 (8.0–10.3)	11.7 (11.2–12.2)
	Inhalants within lifetime	3.6 (2.7–4.4)	7.8 (7.0–8.6)	3.0 (2.3–3.6)	5.5 (4.6–6.4)	7.4 (6.3–8.4)	5.5 (5.1–5.9)
	Cannabis use within lifetime	12.4 (10.9–14.0)	8.5 (7.6–9.3)	7.4 (6.4–8.4)	6.1 (5.2–7.0)	6.6 (5.7–7.6)	8.0 (7.5–8.4)
	Tranquilliser or sedative use within lifetime	2.3 (1.6–3.1)	5.8 (5.1–6.5)	5.4 (4.6–6.3)	5.8 (4.9–6.8)	6.9 (5.9–7.9)	5.5 (5.1–75.9)
Gambling	Slots within last 12 months	2.6 (1.9–3.4)	19.6 (18.4–20.8)	3.7 (3.0–4.4)	1.4 (0.9–1.8)	4.4 (3.6–5.2)	8.0 (7.6–8.5)
	Cards within last 12 months	5.6 (4.5–6.7)	8.3 (7.4–9.1)	3.5 (2.8–4.2)	6.7 (5.8–7.7)	7.2 (6.2–8.2)	6.5 (6.1–6.9)
	Lottery within last 12 months	10.3 (8.8–11.8)	12.2 (11.2–13.2)	13.6 (12.3–14.9)	10.0 (8.8–11.1)	8.3 (7.2–9.3)	11.1 (10.5–11.6)
	Sports betting within last 12 months	10.6 (9.1–12.1)	8.7 (7.8–9.6)	5.8 (4.9–6.7)	3.8 (3.0–4.5)	6.7 (5.8–7.7)	7.1 (6.6–7.5)
	Online within last 12 months	15.6 (13.9–17.4)	14.6 (13.5–15.7)	11.5 (10.3–12.7)	9.1 (8.0–10.2)	10.3 (9.1–11.5)	12.2 (11.7–12.8)
	Gambling total within last 12 months	21.5 (19.4–23.4)	28.5 (27.1–29.9)	18.6 (17.0–20.0)	16.1 (14.6–17.5)	16.6 (15.1–18.0)	22.0 (21.0–23.0)

Data shown are proportions with 95% confidence intervals by country.

### Measurements

Data on problem gambling were only available for Denmark, Finland and Sweden. Data regarding all other variables were available for all Nordic countries.

#### Past year gambling participation

Gambling participation was measured by participation in four gambling types: slot machines, card or dice games, lotteries and sports betting. A dichotomised variable for overall gambling was computed based on participation in the four gambling types in the last 12 months. Online gambling was computed as an additional variable.

#### Problem gambling

The ESPAD survey used the Lie/Bet questionnaire, which is presumably applicable to adolescents [34,35] and comprises two ‘yes or no’ questions: ‘Have you ever had to lie to people important to you about how much you gambled?’ and ‘Have you ever felt the need to bet more and more money?’ As suggested in earlier work [35], a positive answer to at least one of those questions determines a status of probable problem gambling.

#### Leisure activities and school truancy

Reports of gaming too much, sports participation and reading for enjoyment were coded as ‘almost every day/less than once a week’ versus ‘never/a few times a year/once or twice a month’. School truancy was measured as how many days the person missed one or more classes in the last 30 days. The cut-off for the dichotomised variable was ‘missed three days or more’.

#### Parental relationships

Family environment was operationalised into whether students could easily obtain money as a gift from their parents, parental control and parental emotional support. All variables were coded into the dichotomised categories. Parental control was measured as parents’ monitoring of Saturday night activities and coded into ‘usually do not know/know sometimes’ versus ‘know quite often/know always’. Whether the students could easily get emotional support or money as gift from their mother and/or their father was coded into the categories ‘almost never/seldom/sometimes’ versus ‘almost always/often’.

#### Substance use

Questions on alcohol use and binge drinking were limited to within the last month, while those on other substance use referred to the student’s lifetime. All variables were coded ‘yes’ or ‘no’. Binge drinking was defined as having been intoxicated from alcoholic beverages, for example staggering when walking, not being able to speak fluently or vomiting.

### Statistical analysis

First, the prevalence for gambling and problem gambling for each country in the Nordic region was measured by proportions with 95% confidence intervals (CI). Further, reported rates for variables of leisure, school truancy, family environment and substance use were presented with 95% CI. This was presented by each country and for the Nordic countries in general.

Second, the variables were tested for problem gambling by bivariate logistic regression. All factors significantly associated with problem gambling were used in the multivariate logistic regression modelling. The first model examined factors regarding leisure, school truancy and family environment. The variables concerning substance use were tested in model 2 and gambling of different types in model 3. The factors still showing significance were tested in a final model. Both the bivariate and multivariate analyses were controlled by sex and presented as odds ratios (OR) and 95% CI. The procedure was repeated for each country that participated in the option to report problem gambling (Denmark, Finland and Sweden). It should be noted that the statistical power is higher in the analyses collapsed for all countries compared to analyses conducted separately for each country. All analyses included both gamblers and non-gamblers. Further, all statistical tests were two-sided, and a *p*-value of ⩽0.05 was considered to be statistically significant.

Missing responses varied between 1.0% (Denmark) to 5.1% (Sweden) for gambling and between 3.2% (Finland) to 5.4% (Sweden) for problem gambling. Missing responses were not included in analyses.

All analyses were conducted using IBM SPSS Statistics for Windows v22 (IBM Corp., Armonk, NY).

## Results

### Problem gambling in Denmark, Finland and Sweden

Sweden had the lowest rate of problem gambling (5.4%; 95% CI 4.5–6.3). There was no significant difference between Denmark (8.6%; 95% CI 7.3–10.0) and Finland (9.0%; 95% CI 8.1–9.9; Table I).

### Gambling in all the Nordic countries

Finland had a significantly higher overall gambling rate (28.5%; 95% CI 27.1–29.9) than other Nordic countries (average 7.8%; 95% CI 7.8–8.4). The gambling prevalence ranged from 16.1% (Norway) to 28.5% (Finland). The proportion of gambling was lower in Iceland, Norway and Sweden than it was in Denmark and Finland. The lottery was the most common gambling type in the Nordic region (11.1%; 95% CI 10.5–11.6). Adolescents who had gambled online constituted 12.2% (95% CI 11.7–12.8). Gambling on slot machines (8.0%; 95% CI 7.6–8.5) was slightly more common than both cards (6.5%; 95% CI 6.1–6.9) and betting (7.1%; 95% CI 6.6–7.5). One in eight students had gambled online during the previous year. The proportion was lower in Iceland, Norway and Sweden compared to Denmark and Finland. Norway and Sweden had a lower prevalence than Iceland.

### Leisure activities, school truancy, family environment and substance use in all Nordic countries

Table I shows that most adolescents reported high levels of parental control (91.3%; 95% CI 90.8–91.2) and emotional support (88.0%; 95% CI 87.8–88.8). Seventeen per cent (95% CI 16.4–17.7) could easily obtain money as a gift from their parents. The majority also reported active participation in sports (89.7%; 95% CI 89.2–90.2). Reading books for enjoyment was done by 22.8% (95% CI 22.1–23.5), while 18.2% (95% CI 17.6–18.9) reported gaming too much.

The Nordic average for alcohol consumption over the last month was 29.9% (95% CI 29.1–30.7), and 11.7% reported binge drinking in the last month (95% CI 11.2–12.2). There were large variations between countries in the variables. The average lifetime prevalence rate for cannabis, inhalants and tranquillisers or sedatives were all within 5–8% (Table I).

### Covariates to problem gambling in Denmark, Finland and Sweden

Table II shows that all gambling types were correlated with problem gambling in the bivariate analyses for all countries available for analyses (Denmark, Finland and Sweden). Further, except for participation in sports, alcohol use in the last month and missing classes, all other factors were found to be significant. Reading books for enjoyment (OR=0.5, *p*=0.000) and parental control (OR=0.4, *p*=0.000) were negatively associated with problem gambling (Table II).

**Table II.** Bivariate analyses and three-step multivariate analyses on associations with problem gambling for Denmark, Finland and Sweden combined. All analyses were controlled by sex.

		Bivariate (OR)	Model 1	Model 2	Model 3	Final model
Leisure activities	Spend too much time gaming	2.6 (2.2–3.2)***	2.7 (2.2–3.2)***			2.7 (2.3–3.4)***
	Active participation in sports	1.0 (0.8–1.4) ns				
	Read books for enjoyment	0.5 (0.4–0.6)***	0.5 (0.4–0.7)***			0.7 (0.4–1.4) ns
School truancy	3 or more days of school missed	1.1 (0.9–1.5) ns				
Relationship with parents	Can easily obtain money as a gift from parents	1.2 (0.7–1.9) ns				
	Parental monitoring of Saturday night activities	0.4 (0.3–0.5)***	0.4 (0.3–0.5)***			0.7 (0.5–0.9)***
	Parental emotional support	0.6 (0.5–0.7)***	0.7 (0.6–0.9**			0.8 (0.6–1.0)*
Substance use	Alcohol use within last month	1.4 (0.9–2.0) ns				
	Binge drinking within last month	2.2 (1.3–3.7)**		1.5 (1.2–1.9)**		1.2 (0.9–1.5) ns
	Inhalants within lifetime	4.4 (2.9–7.0)***		4.0 (2.4–6.5)***		1.7 (1.2–2.2)**
	Cannabis use within lifetime	1.8 (1.0–3.3)*		1.4 (1.0–1.8)*		1.0 (0.7–1.4) ns
	Tranquilliser or sedative use within lifetime	2.4 (1.3–4.3)**		1.2 (0.8–3.0) ns		
Gambling	Gambling: slots within last 12 months	7.5 (4.7–12.1)***			2.6 (2.1–3.2)***	2.8 (2.3–3.6)***
	Gambling: cards within last 12 months	4.7 (3.1–7.5)***			1.4 (1.1–1.8)**	1.3 (1.0–1.7) ns
	Gambling: lottery within last 12 months	4.4 (2.9–6.8)***			1.1 (0.9–1.3) ns	
	Gambling: sports betting within last 12 months	5.4 (3.5–8.2)***			2.4 (1.9–3.0)***	2.3 (1.7–2.9)***
	Gambling: online within last 12 months	5.0 (3.4–7.4)***			2.5 (1.9–3.1)***	2.3 (1.8–3.0)***

* *p*<0.05; **p<0.01; ***p<0.000.

OR: odds ratio.

In the final model, the remaining significant predictors were reporting spending too much time gaming (OR=2.7, *p*=0.000), parental control (OR=0.7, *p*=0.000), parental emotional support (OR=0.8, *p*=0.05) and inhalants (OR=1.7, *p*=0.01). The gambling types that remained significant were slots (OR=2.8, *p*=0.000), betting (OR=2.3, *p*=0.000) and online gambling (OR=2.3, *p*=0.000). Parental emotional support was only significant in this merged analysis and not for any individual country.

#### Denmark

Adolescents in Denmark showed higher rates of parental control (96.3%; 95% CI 88.4–91.3) compared to the Nordic average (91.3%; 95% CI 90.8–91.2) but were more likely to report obtaining money easily as a gift from parents (21.0%; 95% CI 19.9–22.9; Table II). Danish adolescents were less likely to miss school (25.8%; 95% CI 13.6–27.9) or have used inhalants or tranquillisers/sedatives in their lifetime (2.3%; 95% CI 1.6–3.1). However, compared to adolescents in other Nordic countries, they were dramatically more likely to have consumed alcohol (73.5%; 95% CI 71.4–75.6), to have gone on drinking binges (31.9%; 95% CI 29.6–34.1) and to have used cannabis (12.4%; 95% CI 10.9–14.0). The proportion who had participated in sports, read books or spent too much time gaming did not differ from the Nordic average. Denmark had a high rate of sports betting (10.6%; 95% CI 9.1–12.1) compared to other Nordic countries except Finland (8.7%; 95% CI 7.8–9.6). Gambling on cards was less common in Denmark (5.6%; 95% CI 4.5–6.7) compared to Finland (8.3%; 95% CI 7.4–9.1) but not compared to Norway (6.7%; 95% CI 5.8–7.7) and Sweden (7.2%; 95% CI 6.2–8.2). As shown in Table III, the relationship with parents, including parental monitoring, was singled out as a strong factor in Denmark. Further, not obtaining money easily from parents was negatively associated with problem gambling. No leisure activity was associated with problem gambling, even in the bivariate analyses. Binge drinking and inhalants were associated in the second model, but did not reach significance in the final model. Gambling on slots was the only gambling type correlated with problem gambling in the final model (OR=3.7, *p*=001).

**Table III.** Bivariate analyses and three-step multivariate analyses on associations with problem gambling in Denmark. All analyses controlled by sex.

		Bivariate (OR) controlled by sex	Model 1	Model 2	Model 3	Final model
Leisure activities	Spend too much time gaming	1.3 (0.9–2.0) ns				
	Active participation in sports	1.4 (0.7–2.9) ns				
	Read books for enjoyment	0.7 (0.4–1.1) ns				
School truancy	3 or more days of school missed	1.1 (0.7–1.7) ns				
Relationship with parents	Can easily obtain money as a gift from parents	3.5 (1.9–6.4)***		3.7 (2.0–6.7)***		3.4 (1.8–6.3)***
	Parental monitoring of Saturday night activities	0.3 (0.1–0.5)***		0.2 (0.1–0.4)***		0.3 (0.1–0.6)***
	Parental emotional support	0.7 (0.4–1.1) ns				
Substance use	Alcohol use within last month	1.3 (0.8–1.9) ns				
	Binge drinking within last month	1.7 (1.2–2.5)**		1.5 (1.0–2.2)*		1.3 (0.9–2.0) ns
	Inhalants within lifetime	2.9 (1.5–5.9)**		2.7 (1.3–5.5)**		1.4 (0.9–2.1) ns
	Cannabis use within lifetime	1.3 (0.8–2.2) ns				
	Tranquilliser or sedative use within lifetime	2.3 (0.8–6.4) ns				
Gambling	Gambling: slots within last 12 months	5.7 (2.8–11.6)***			3.2 (1.8–5.8)***	3.7 (1.6–8.5)**
	Gambling: cards within last 12 months	3.0 (1.8–5.3)***			2.3 (1.0–5.2)*	1.7 (0.9–3.2) ns
	Gambling: lottery within last 12 months	3.4 (2.2–5.2)***			0.9 (0.5–1.7) ns	
	Gambling: sports betting within last 12 months	4.0 (3.1–5.2)***			1.5 (0.8–2.5) ns	
	Gambling: online within last 12 months	5.3 (3.7–7.8)***			1.6 (0.9–2.8) ns	

* *p*<0.05; **p<0.01; ***p<0.001.

#### Finland

Adolescents in Finland had a high level of sports participation (95.3%; 95% CI 94.7–96.0; Table I), and had used inhalants (87.8%; 95% CI 7.0–8.6) and missed school (38.9%; 95% CI 37.3–40.4) to a higher degree than adolescents in other Nordic countries. Parental monitoring was significantly lower (88.0%; 95% CI 87.0–89.0), as was as the proportion of youth who read books for enjoyment (18.3%; 95% CI 17.1–19.5). They did not differ from the Nordic averages in reporting gaming too much, drinking alcohol, binge drinking and use of cannabis or tranquillisers/sedatives. One striking difference was the significantly higher level of gambling on slot machines in Finland (19.6%; 95% CI 18.4–20.8) than in the other Nordic countries. Norway had the lowest level (1.4%; 95% CI 0.9–1.8). The lottery seemed slightly more popular in Finland (and Iceland) among adolescents compared to Denmark, Norway and Sweden (Table I). As stated earlier, sports betting was as common in Finland as in Denmark, while the proportion that gambled on card games was larger. In relation to problem gambling, all gambling types besides the lottery, as well as gaming too much and parental monitoring, were significant in the final model (Table IV). Alcohol and other substances were not significant in the final model.

**Table IV.** Bivariate analyses and three-step multivariate analyses on associations with problem gambling in Finland. All analyses controlled by sex.

		Bivariate (OR)	Model 1	Model 2	Model 3	Final model
Leisure	Spend too much time gaming	1.8 (1.4–2.3)***	3.2 (2.5–40)***			3.3 (2.5–4.3)***
	Active participation in sports	0.8 (0.5–1.3) ns				
	Read books for enjoyment	0.4 (0.3–0.6)***	0.6 (0.4–0.8)**			0.8 (0.6–1.2) ns
School truancy	3 or more days of school missed	1.1 (0.9–1.4) ns				
Relationship with parents	Can easily obtain money as a gift from parents	1.2 (0.9–1.6) ns				
	Parental monitoring of Saturday night activities	0.4 (0.3–0.5)***	0.4 (0.3–0.5)***			0.6 (0.4–0.8)**
	Parental emotional support	0.6 (0.5–0.8)**	0.7 (0.5–1.0)*			0.7 (0.5–1.0) ns
Substance use	Alcohol use in last month	1.6 (1.2–2.0)***		1.2 (0.9–1.5) ns		
	Binge drinking in last month	1.9 (1.4–2.5)***		1.3 (0.9–1.5) ns		
	Inhalants within lifetime	2.8 (2.0–3.9)***		2.0 (1.4–2.9)***		1.4 (0.9–2.1) ns
	Cannabis use within lifetime	2.0 (1.5–2.8)***		1.3 (0.9–1.9) ns		
	Tranquilliser or sedative use within lifetime	3.2 (2.2–4.9)***		2.0 (1.2–3.2)***		1.6 (1.0–2.6) ns
Gambling	Gambling: slots within last 12 months	3.8 (3.0–4.9)***			2.2 (1.6–2.9)***	3.1 (2.3–4.2)***
	Gambling: cards within last 12 months	3.4 (2.6–4.5)***			1.6 (1.2–2.3)**	1.6 (1.1–2.2)**
	Gambling: lottery within last 12 months	2.9 (2.2–3.8)***			1.1 (0.8–1.5) ns	
	Gambling: sports betting within last 12 months	4.0 (3.1–5.2)***			1.7 (1.2–2.4)**	2.2 (1.5–3.0)***
	Gambling: online within last 12 months	4.1 (3.2–5.3)***			1.8 (1.3–2.5)***	1.7 (1.2–2.3)**

* *p*<0.05; **p<0.01; ***p<0.001.

#### Sweden

The proportion of adolescents reporting spending too much time gaming in Sweden was above the Nordic average (23.7%; 95% CI 22.0–25.4 compared to 18.2%; 95% CI 17.6–18.9; Table I). Parental monitoring was lower than average, as were alcohol use and binge drinking. Swedish adolescents met the Nordic average concerning participation in sports, reading books, missing school and cannabis use. Further, the proportion of adolescents reporting obtaining money easily as a gift met the Nordic average. Sweden had the lowest rate of lottery participation among youth (8.3%; 95% CI 7.2–9.3). Sports betting was lower in Sweden (6.7%; 95% CI 5.8–7.7) compared to Denmark and Finland but higher than in Norway. Gambling on slots was much lower (4.4%; 95% CI 3.6–5.2) than in Finland, although higher than in Denmark and Norway. Regarding card games, Sweden did not differ from Finland and Denmark. As shown in Table V, reporting gaming too much was significant in the final model (OR=2.7, *p*=0.001). In addition, lifetime use of inhalants (OR=3.4, *p*=0.000), gambling on slot machines (OR=3.6, *p*=0.000), betting (OR=2.3, *p*=0.001) and online gambling (OR=1.9, *p*=0.05) were significant.

**Table V.** Bivariate analyses and three-step multivariate analyses on associations with problem gambling in Sweden. All analyses controlled by sex.

		Bivariate (OR)	Model 1	Model 2	Model 3	Final model
Leisure	Spend too much time gaming	2.1 (1.4–2.9)***	2.8 (1.8–4.2)***			2.7 (1.8–4.0)**
	Active participation in sports	0.7 (0.4–1.2) ns				
	Read books for enjoyment	0.6 (0.4–1.1) ns				
School truancy	3 or more days of school missed	1.6 (1.1–2.4)*	1.4 (0.9–2.0) ns			
Relationship with parents	Can easily obtain money as a gift from parents	1.2 (0.7–1.9) ns				
	Parental monitoring of Saturday night activities	0.5 (0.3–0.8)**	0.5 (0.3–0.9)*			0.9 (0.5–1.6) ns
	Parental emotional support	0.4 (0.3–0.6)***	0.5 (0.3–0.8)**			0.7 (0.4–1.1) ns
Substance use	Alcohol use within last month	1.4 (0.9–2.0) ns				
	Binge drinking within last month	2.2 (1.3–3.7)**		1.8 (1.0–3.2) ns		
	Inhalants within lifetime	4.4 (2.9–7.0)***		4.0 (2.4–6.5)***		3.4 (1.9–6.0)***
	Cannabis use within lifetime	1.8 (1.0–3.3)*		0.9 (0.4–1.8) ns		
	Tranquilliser or sedative use within lifetime	2.4 (1.3–4.3)**		1.8 (1.0–3.5) ns		
Gambling	Gambling: slots within last 12 months	7.5 (4.7–12.1)***			2.9 (1.6–5.4)**	3.6 (1.9–6.7)***
	Gambling: cards within last 12 months	4.7 (3.1–7.5)***			1.2 (0.7–2.4) ns	
	Gambling: lottery within last 12 months	4.4 (2.9–6.8)***			1.4 (0.7–2.5) ns	
	Gambling: sports betting within last 12 months	5.4 (3.5–8.2)***			2.4 (1.3–4.4)**	2.3 (1.2–4.4)**
	Gambling: online within last 12 months	5.0 (3.4–7.4)***			2.3 (1.3–4.1)*	1.9 (1.1–3.5)*

* *p*<0.05; **p<0.01; ***p<0.001.

## Discussion

Cross-country differences were found in gambling between the Nordic countries. Cross-country differences in problem gambling were also found between Denmark, Finland and Sweden, the Nordic countries that participated in the optional section in the ESPAD 2015 regarding problem gambling. Contrary to earlier findings [20], our results showed a lower proportion of problem gambling among adolescents in Sweden than in Denmark and Finland, the only two Nordic countries participating in the ESPAD in both 2011 and 2015. There was no significant difference between Denmark and Finland in 2015 mainly due to a decreasing rate of problem gambling in Finland. The introduction of age restrictions in Finland seems to have decreased problem gambling among youth significantly, which implies that a minimum gambling age of 18 is an effective harm minimisation measure [29,36]. This presumed effect is coherent with the decrease in slot machine use in Norway when Norway raised the legal age from 16 to 18 [37]. Further, Norway has worked on decreasing risks by removing slot machines and replacing them with less attractive devices. Norway respondents did not participate in the optional section on problem gambling, but their gambling rate for slots was the lowest in the Nordic region. Finland still had a higher rate of gambling on slot machines than any other country in 2015. Slots are widely available both online and in real life, in casinos and kiosks, restaurants, gas stations and shopping centres [5]. The prevalence of online gambling was lower in Iceland, Norway and Sweden, which had a more restrictive approach at the time.

According to Jensen, the variations between the Nordic countries are more likely to be due to national differences in gambling preferences, access to games and public policies concerning gambling than to interventionist traditions [25]. Our findings support this notion. The differences in participation in different gambling types seemed to be in line with gambling policies.

The differences in the prevalence of cannabis use and alcohol consumption between the countries also indicated the importance of policies. Denmark had dramatically higher rates of cannabis use, alcohol consumption and binge drinking in the last month than the other countries had. Drug strategies related to cannabis look different in different Nordic countries [38]. All included countries, except for Denmark, are influenced by the vision of a drug-free society. Denmark has historically focused less on consumption and more on criminalising the sale of drugs. Consumption is not controlled, either by law or through official guidelines [38]. Alcohol advertisements are not allowed to target younger people in the Nordic countries. In Sweden, ‘younger’ is defined as <25 years, and in Denmark it is defined as <18 years [39]. In Iceland, all kinds of alcohol advertisements are forbidden, and the age limit for both on- and off-premise sales is 20 years. For all other countries, people aged ⩾18 can buy alcohol (although some countries have a limit of >20 for stronger beverages or off-premises sale) [39]. Iceland had significantly lower rates than the other countries for the variables for alcohol.

Cannabis use and alcohol consumption were not associated with problem gambling in the bivariate analyses in Denmark. In Finland, both were significant, while in Sweden, cannabis use acted as a predictor for problem gambling in the bivariate analyses. The differences may be due to the high rate in Denmark.

Regarding problem gambling, few covariates remained significant in the final models. Slots were the only gambling type that was significant for all countries and in the merged analysis. In general, gaming too much, inhalants, slots, betting and online gambling had a positive relationship with problem gambling. Parental monitoring and parental emotional support acted as potential protective factors, since they were significant negatively predictors. This is in line with earlier findings [9]. For example, Parrado-Gonzalez and Léon-Jariego found that for adolescents with high family support, exposure to gambling advertising did not promote favourable attitudes towards gambling, and gambling frequency had less of an effect on problem gambling [40]. None of the variables related to parents was significant in Sweden, while parental monitoring was related to problem gambling in Denmark and Finland. Although significant in the analysis for all countries, inhalants were only associated with problem gambling in Sweden when controlling for other variables. The association with different alcohol and other substance use was not that evident in the final model for problem gambling. This is in line with the results of a Swedish school survey [41].

Participation in sports was not associated with problem gambling in the bivariate analysis for the included countries in general or for any one country individually. This contradicts earlier work [42]. Further, similar results were found for school truancy and alcohol use except that school truancy was significant for Sweden and alcohol use was significant for Finland in the bivariate analyses.

As shown, the covariates that were found to be significant in the common model for Denmark, Finland and Sweden were not associated with problem gambling in those countries. In addition, earlier research has shown that perceptions of addictions, gambling included, vary between the Nordic countries [43]. For example, Swedes seemed overall to be more concerned and Finns less concerned than others about addiction as a severe societal problem. More specifically, Swedes and Norwegians rated addiction to ‘hard’ drugs as more serious than other addiction problems, whereas Finns reserved that rating for addiction to alcohol. In addition, Finns seemed to have a stronger belief than others that people are able to solve such problems without professional treatment. In line with this, Finns also seem to take a more ‘moral’ view than others towards addiction (not least to alcohol), in attributing the responsibility for acquiring and solving these problems largely to the individual, whereas Swedes seemed more inclined to see other circumstances as responsible.

## Conclusions

One main conclusion of our study is that universal models for factors associated with problem gambling have shortcomings. Further, youth’s community contexts should be considered in research, policy and interventions.

### Limitations

The data are cross-sectional. To be more certain about causality, longitudinal data and time series analyses are required. Furthermore, our analyses extend from survey data with known limitations and shortcomings. Norway and Iceland did not take part in the optional questions on problem gambling. This is unfortunate, since they have adopted a more restrictive approach to gambling regulation, and comparisons would be valuable. In addition, in abandoning universal approaches, using separate models for sex may give important insights. Although an important factor in youth problem gambling, mental health is not included in the ESPAD and therefore is missing in our analyses.

Cultural contexts are important in cross-country comparisons. Cultural context may affect the interpretations and answers from respondents. However, as part of the preparations for the ESPAD 1999, a methodological study was conducted to ascertain better the role of cultural context in different countries. Data were collected in seven countries in different parts of Europe, and the study showed that both reliability and validity were high in all countries. The study indicates that the influence of the cultural context in regard to the ESPAD questionnaire could be rather limited.

This study is based on secondary data from the ESPAD, one of the largest cross-country surveys in the world. All samples are nationally representative. In spite of the efforts of the ESPAD team to make the data comparable, there are differences in the included countries that should be considered when interpreting the results. The response rate from schools was low in Denmark. There were no indications of bias noted for the net sample though, and the Danish team found the collected data to be representative for Danish students. Iceland used a total sample strategy, while the other countries used a strategic random sampling method. Data from Norway were weighted, while all other countries used unweighted data.

It is possible that assessment of problem gambling by use of a short instrument such as the Lie/Bet inflated estimates. Furthermore, the comparability of assessments based on Lie/Bet and other instruments may be hampered by different time frames (i.e. the past 12 months vs. one’s lifetime). However, validations of Lie/Bet indicate that the screen may be useful to assess at-risk gambling in comprehensive youth surveys with a broad range of topics [35]. Further, several areas of interest are measured by single items. For simple (one-dimensional) or concrete constructs that are well understood, such as consumption of certain substances or active participation in sports, a single item may suffice. Complex areas with several dimensions, such as emotional support from parents, could be more problematic. Single items do not capture the construct (low content validity), have fewer points of discrimination (sensitivity) and lack a measure of internal consistency reliability (reliability). In addition, the results may be influenced by the common method bias, creating inflation regarding the relationship between study variables.

### Future direction

Leisure activities, parents, alcohol and drug consumption, school environment, friends, access to gambling and culture are important factors for adolescent problem gambling [44]. More findings are needed on how adolescents experience those factors, and how interactions with them may differ between groups and countries. Like Kristiansen et al., we recommend further focus on the social contexts of adolescent gambling in order to investigate various aspects of gambling in modern youth culture [45]. More cross-country surveys are needed, as well as the participation of all Nordic countries in surveys such as the ESPAD and the optional questions on problem gambling. Qualitative research is needed in order to gain knowledge about the processes and experiences of the youth gambling context in different countries, and in order to understand the effect of different legislation and intervention on underage gambling.

## References

[1] Abbott M Binde P Hodgins D , et al. Conceptual framework of harmful gambling: an international collaboration. Guelph: Ontario Problem Gambling Research Centre (OPCRC), 2013.

[2] Langham E Thorne H Browne M , et al. Understanding gambling related harm: a proposed definition, conceptual framework, and taxonomy of harms. BMC Public Health 2015;16:1–2. 10.1186/s12889-016-2747-0 PMC4728872 26818137

[3] Lorains K Cowlishaw S Thomas A. Prevalence of comorbid disorders in problem and pathological gambling. Systematic review and meta-analysis of population surveys. Addiction 2015;106:490–8. 10.1111/j.1360-0443.2010.03300.x 21210880

[4] Dowling N Cowlinshaw S Jackson A , et al. The prevalence of comorbid personality disorders in treatment-seeking problem gamblers. A systematic review and meta-analysis. J Pers Disord 2015;29:515–39. 10.1521/pedi_2014_28_168 25248010

[5] Salonen A Hellman M Latvala T , et al. Gambling participation, gambling habits, gambling-related harm, and opinions on gambling advertising in Finland in 2016. Nord Stud Alcohol Dr 2018;35:215–34. 10.1177/1455072518765875 PMC7434154 32934528

[6] Dussault F Brendgen M Vitaro F , et al. Longitudinal links between impulsivity, gambling problems and depressive symptoms: a transactional model from adolescence to early adulthood. J Child Psychol Psychiatry 2011;52:130–8. 10.1111/j.1469-7610.2010.02313.x 20854365

[7] Blinn-Pike L Worthy SL Jonkman JN. Adolescent gambling: a review of an emerging field of research. J Adolesc Health 2010;47:223–36. 10.1016/j.jadohealth.2010.05.003 20708560

[8] Kryszajtys D Hahmann T Schuler A , et al. Problem gambling and delinquent behaviours among adolescents: a scoping review. J Gambl Stud 2018;34:893–914. 29470759 10.1007/s10899-018-9754-2 PMC6096515

[9] Dowling N Merkouris S Greenwood C , et al. Early risk and protective factors for problem gambling: a systematic review and meta-analysis of longitudinal studies. Clin Psychol Rev 2017;51:109–24. 10.1016/j.cpr.2016.10.008 27855334

[10] Carbonneau R Vitaro F Brendgen M , et al. Variety of gambling activities from adolescence to age 30 and association with gambling problems: a 15-year longitudinal study of a general population sample. Addiction 2015;110:1985–93. 10.1111/add.13083 26234317

[11] Kulturdepartementet. Handlingsplan mot spilleproblemer 2019–2021, December 2018. https://www.regjeringen.no/no/dokumenter/handlingsplan-mot-spilleproblemer-2019-2021/id2623296/ (accessed 20 March 2020).

[12] Sundheds og Ældreministeriet. Ludomani i Danmark. Strategi til förebyggelse. February 2018. http://www.sum.dk/Aktuelt/Nyheder/Sundhedspolitik/2018/Februar/~/media/Filer%20-%20Publikationer_i_pdf/2018/Ludomanistrategi/6993_SUM_Ludomani_web.pdf (accessed 20 March 2020).

[13] Järvinen-Tassopoulus J. State of play 2017. A review of gambling in Finland. Helsinki, Finland: National Institute for Health and Welfare, 2018.

[14] Linell A Richardson M Wamala S. The Swedish National Public Health policy report 2010. Scand J Public Health 2013;41:3–56. 10.1177/1403494812466989 23341365

[15] Olason D Gretarsson S. Iceland. In: Meyer G Hayer T Griffiths M (eds) Problem gambling in Europe: challenges, prevention, and interventions. New York: Springer, 2009, pp.137–53.

[16] Calado F Alexandre J Griffiths M. Prevalence of adolescent problem gambling: a systematic review of recent research. J Gambl Stud 2017;33:397–424. 27372832 10.1007/s10899-016-9627-5 PMC5445143

[17] Kristiansen S Jensen S. Prevalence of gambling problems among adolescents in the Nordic countries: an overview of national gambling surveys 1997–2009. Int J Soc Welfare 2011;20:75–86.

[18] Kraus L Guttormsson U Leifman H , et al. ESPAD Report 2015. Results from the European School Survey Project on alcohol and other drugs. ESPAD and EMCDDA. Luxembourg: Publications Office of the European Union, 2016.

[19] Molinaro S Benedetti E Scalese M , et al. Prevalence of youth gambling and potential influence of substance use and other risk factors across 33 European countries: first results from the 2015 ESPAD study. Addiction 2018;113:1862–73. 10.1111/add.14275 29806197

[20] Molinaro S Canale N Vieno A , et al. Country- and individual-level determinants of probable problematic gambling in adolescence: a multi-level cross-national comparison. Addiction 2014;109:2089–97. 10.1111/add.12719 25171305

[21] Hellman M. How are vulnerable groups affected by social and health care reforms? Nord Stud Alcohol Dr 2019;36:3–5. 10.1177/1455072519829392 PMC7434168 32934545

[22] Viner RM Ozer EM Denny S , et al. Adolescence and the social determinants of health. Lancet 2012;379:1641–52. 10.1016/S0140-6736(12)60149-4 22538179

[23] Balaj M Huijts T McNamara CL , et al. Non-communicable diseases and the social determinants of health in the Nordic countries: findings from the European Social Survey (2014) special module on the social determinants of health. Scand J Public Health 2017;45:90–102. 28128015 10.1177/1403494816686026

[24] Nikkinen JT Egerer MD Marionneau VK. Introduction. In: Egerer M Marionneau V Nikkinen J (eds) Gambling policies in European welfare states: current challenges and future prospects. Basingstoke, UK: Palgrave Macmillan, 2018, pp.1–14.

[25] Jensen C. Money over misery. Restrictive gambling legislation in an era of liberalization. J Eur Public Policy 2017;24:1–16.

[26] Cisneros Örnberg J Hettne J . The future Swedish gambling market. Challenges in the law and public policies. In: Egerer M Marionneau V Nikkinen J (eds) Gambling policies in European welfare states: current challenges and future prospects. Basingstoke: Palgrave Macmillan, 2018, pp.297–314.

[27] Hanss D Mentzoni R Blaszcynski A , et al. Prevalence and correlates of problem gambling in a representative sample of Norwegian 17-year-olds. J Gambl Stud 2015;31:659–78. 10.1007/s10899-014-9455-4 PMC4534503 24619792

[28] Jonsson J. Preventing problem gambling: focus on overconsumption. PhD dissertation, Stockholm University, Sweden, 2019.

[29] Nordmyr J Österman K. Raising the legal gambling age in Finland: problem gambling prevalence rates in different age groups among past-year gamblers pre- and post-implementation. Int Gambl Stud 2016;16:347–56.

[30] Government of Iceland. Lotteries, https://www.government.is/topics/consumer-affairs/lotteries/ (accessed 19 March 2019).

[31] Olason D Kristjansdottir E Einarsdottir H , et al. Internet gambling and problem gambling among 13 to 18 year old adolescents in Iceland. Int J Mental Health Addict 2010;9:257–63.

[32] Dickson L Derevensky J Gupta R. Youth gambling problems: examining risk and protective factors. Int Gambl Stud 2008;8:25–47.

[33] European Monitoring Centre for Drugs and Drug Addiction. ESPAD 2015 methodology, http://espad.org/sites/espad.org/files/TD0116477ENN_002.pdf (accessed 27 March 2020).

[34] Götestam KG Johansson A Wenzel HG , et al. Validation of the Lie/Bet screen for pathological gambling on two normal population data sets. Psychol Rep 2004;95:1009–13. 10.2466/pr0.95.3.1009-1013 15666948

[35] Rossow I Molde H. Chasing the criteria: comparing SOGS-RA and the Lie/Bet screen to assess prevalence of problem gambling and ‘at-risk’ gambling among adolescents. J Gambl Issues 2006;18:57–71.

[36] Raisamo S Warpenius K Rimpela A. Changes in minors gambling on slot machines in Finland after the raising of the minimum legal gambling age from 15 to 18 years. A repeated cross-sectional study. Nord Stud Alcohol Dr 2015;32:579–90.

[37] Rossow I Bang Hansen M Storvoll E. Changes in youth gambling after the removal of slot machines in Norway. Nord Stud Alcohol Dr 2013;30:317–30.

[38] Egnell S Villman E Obstbaum Y. Cannabis policy and legislation in the Nordic countries. A report on the control of cannabis use and possession in the Nordic legal systems. Stockholm: Nordic Welfare Centre, 2019.

[39] Häkkinen E. Information on the Nordic alcohol market 2019. Helsinki: Alko, Inc., 2019.

[40] Parrado Parrado-González A Léon-Jariego J . Exposure to gambling advertising and adolescent gambling behavior. Moderating effects of perceived family support. Int Gambl Stud. Epub ahead of print 11 Jan 2020. DOI: 10.1080/14459795.2020.1712737.

[41] Svensson J Sundqvist K. Gambling among Swedish youth: predictors and prevalence among 15- and 17-year-old students. Nord Stud Alcohol Dr 2019;36:177–89. 10.1177/1455072518807788 PMC7434126 32934558

[42] Stillman MA Brown T Ritvo EC , et al. Sport psychiatry and psychotherapeutic intervention, 2016. Int Rev Psychiatry 2016;28:614–22. 10.1080/09540261.2016.1202812 27683966

[43] Blomqvist J Raitasalo K Melberg H . Popular images of addiction in five European countries. In: Hellman M Berridge V Duke K , et al. (eds) Concepts of addictive substances and behaviours across time and place. Oxford: Oxford University Press, 2016, pp.1–14.

[44] Räsänen T Lintonen T Tolvanen A , et al. The role of social support in association between gambling, poor health and health risk-taking. Scand J Public Health 2016;44:593–8. 10.1177/1403494816654380 27313169

[45] Kristiansen S Trabjerg M Reith G. Learning to gamble: early gambling experiences among young people in Denmark. J Youth Stud 2014;18:133–50.

